# Youth Characteristics Associated With Sexual Violence Perpetration Among Transgender Boys and Girls, Cisgender Boys and Girls, and Nonbinary Youth

**DOI:** 10.1001/jamanetworkopen.2022.15863

**Published:** 2022-06-02

**Authors:** Michele L. Ybarra, Kimberly L. Goodman, Elizabeth Saewyc, Jillian R. Scheer, Ida F. Stroem

**Affiliations:** 1 Center for Innovative Public Health Research, San Clemente, California; 2 Rape, Abuse and Incest National Network, Washington, District of Columbia; 3 Stigma and Resilience Among Vulnerable Youth Centre, School of Nursing, University of British Columbia, Vancouver, British Columbia, Canada; 4 Department of Psychology, Syracuse University, Syracuse, New York; 5 Norwegian Centre for Violence and Traumatic Stress Studies, Oslo, Norway

## Abstract

**Importance:**

Gender minority adults experience higher rates of sexual violence (SV) than cisgender adults. How this disparity extends to youths, including perpetration rates, is unknown.

**Objective:**

To compare rates of experience and perpetration of SV by gender identity and investigate characteristics associated with odds of perpetration within gender identity categories.

**Design, Setting, and Participants:**

This cross-sectional study used baseline data from a national online longitudinal survey collected in 2018 to 2020. Eligible participants were youths ages 14 to 16 years who read English and had internet access. Data were analyzed in November 2021 and March 2022.

**Exposures:**

Youth characteristics included stressors associated with being marginalized (eg, internalized transphobia), general stressors (eg, trauma not associated with experiencing SV), other marginalized statuses (eg, identifying as a girl), factors associated with protection (eg, social support), environmental characteristics (eg, community violence exposure), and risk factors associated with SV (eg, consumption of violent pornography).

**Main Outcomes and Measures:**

Self-reported rates of experiencing and perpetrating SV, defined as sexual assault, rape, attempted rape, and coercive sex, among cisgender, transgender, and nonbinary youths.

**Results:**

Among 4193 youths in the sample (mean [SD] age, 14.8 [0.7] years), 3282 participants (78.3%) were cisgender, 329 participants (7.9%) were transgender, and 582 participants (13.9%) were nonbinary. The odds of SV perpetration were not statistically significantly different for transgender boys and girls (odds ratio [OR], 0.90; 95% CI, 0.57-1.41; *P* = .64) or nonbinary youths (OR, 0.78; 95% CI, 0.54-1.12; *P* = .18) compared with cisgender boys and girls. By contrast, transgender boys and girls (OR, 2.31; 95% CI, 1.83-2.91; *P* < .001) and nonbinary youths (OR, 2.37; 95% CI, 1.98-2.83; *P* < .001) were more than 2-fold as likely as cisgender boys and girls to report experiencing SV. Aggressive behavior was associated with higher odds of SV perpetration for transgender boys and girls (adjusted OR [aOR], 1.87; 95% CI, 0.75-4.65; *P* = .18) and nonbinary youths (aOR, 1.61; 95% CI, 0.78-3.32; *P* = .20). Indications of hostile masculinity were associated with higher odds of SV perpetration among cisgender youths (ie, positive attitudes for boys to engage in rape behaviors: aOR per unit increase in score, 1.15; 95% CI, 1.07-1.25; *P* < .001; sexual dominance: aOR per unit increase in score, 1.03; 95% CI, 1.01-1.04; *P* < .001) but not among transgender or nonbinary youths.

**Conclusion and Relevance:**

These findings may suggest an important foundation for the development of inclusive, research-based SV prevention programs and methods for incorporating gender identity effectively into SV research.

## Key Points

**Question:**

How do rates of experiencing and perpetrating sexual violence vary by gender identity, and what youth characteristics are associated with perpetration within gender identity categories?

**Findings:**

In this cross-sectional study of 4193 youth aged 14 to 16 years, gender minority youth were more likely to report experiencing sexual violence but not more likely to report perpetrating sexual violence. Risk factors associated with perpetrating sexual violence that may be targeted in prevention programs varied across gender identity, specifically cisgender, transgender, and nonbinary identities.

**Meaning:**

These findings suggest that sexual violence prevention programs should address culturally asserted gender roles with cisgender boys and girls and that different target points may be more relevant for transgender boys and girls and nonbinary youths than for cisgender youths.

## Teaser

This cross-sectional study investigates rates of perpetration and experience of sexual violence by sexual and gender identity among youth ages 14 to 16 years.

## Introduction

Sexual violence (SV) is a significant public health issue. Sexual assault costs individuals who experience it an estimated $18 million annually in the US.^1^ Associated lifetime costs of rape, including mental health care, medical care, and lost productivity, are more than $3 billion in the US.^2^ In addition to societal costs, mental and somatic health impacts on the individual can be high.^3^

SV is unevenly distributed in the population. A study^4^ suggests that transgender men and women and other gender minority adults (eg, genderfluid and nonbinary individuals) experience sexual assault and rape at rates 2-fold to 6-fold those of cisgender men and women. Indeed, nearly half (47%) of gender minority individuals experience sexual violence in their lifetimes.^5,6^ Furthermore, studies^7,8^ of gender minority college students found that these individuals had higher levels of SV trauma-related sequalae, including posttraumatic stress disorder, than their cisgender peers.

Given these disparities, efforts to reduce rates of SV among members of gender minority groups are sorely needed but generally lacking. A key part of preventing SV is understanding perpetration. Given that most individuals who sexually aggress appear to do so the first time in adolescence,^9,10,11^ examining perpetration rates in adolescence is warranted.

To contextualize the current study, in 2021, more than 100 legislative bills were proposed in the US to prohibit health care for transgender individuals, restrict facility access based on sex assigned at birth, exclude transgender individuals from athletic activities, and place restrictions on identity documents, among other policies.^12^ This highlights not only the pervasive enacted stigma that gender minority youths face, but also the sensitive and unintentionally political research topic that SV perpetration represents. Public health research should promote equity and inclusion and reduce health disparities experienced by gender minority youths. To this end, we compared SV experience and perpetration rates by gender identity and investigated risk factors associated with perpetration within gender identity categories. Findings may inform how SV perpetration research can be conducted by gender and provide implications for prevention programming.

## Methods

This cross-sectional study was approved by Advarra Institutional Review Board (IRB) and Pearl IRB. A waiver of parental permission was granted by both IRBs to protect participant confidentiality. The response rate, based on the American Association for Public Opinion Research web calculator version 4.1,^13^ ranged from 4171 of 74 193 participants (5.6%; response rate 1) to 5373 of 71 491 participants (7.5%; response rate 4).

Growing Up With Media is a national longitudinal survey of youths designed to investigate SV in adolescence.^14^ The original cohort was recruited in 2006. A new cohort of 4404 youths was recruited between June 2018 and March 2020. The sample size reported here varies from those in previous articles^15,16,17^ because it includes individuals aged 16 years and youths across all recruitment modes.

Most new cohort participants (4152 individuals [94.3%]) were recruited on social media, particularly via advertisements on Facebook and Instagram. A minority of participants were recruited via random digit dial (211 individuals) and address-based sampling (41 individuals) before these strategies were deemed infeasible. Social media ads targeted profiles of youths in the age range (ie, 14-16 years) and encouraged youths to “have your voice heard” and “make a difference.” The survey’s aim, to identify youth characteristics and experiences associated with SV perpetration, was purposefully absent in the advertising to avoid selection bias based on SV-related interest. Targeted advertising language (eg, calling all transgender girls) was used to oversample members of sexual and gender minority groups. Youths who were interested clicked on the online advertisement and were linked to a secure survey website that described the study. Subsequent pages asked eligibility questions. Eligible youths were asked to read and assent to participate before continuing with the main survey.

### Eligible Youth

Eligible youths were living in the US, agreed to provide contact information for follow up, and confirmed their date of birth for verification in subsequent surveys. Initially, we targeted youths aged 14 to 15 years, then expanded to those aged 16 years toward the end of field work to increase age diversity.

Participants received $15. Ineligible youths were directed to a web page that included links to general resources for youths (eg, the Center for Young Women’s Health site^18^). Quotas were used to promote a diverse sample and balance across sex assigned at birth. Once a quota was reached, youths in that bin were no longer eligible.

### Identifying the Analytical Sample

Data were examined for indication (eg, garbled language in open-ended text boxes) of bots (ie, software programs that can automatically or with minimal intervention perform tasks, such as replying to messages); none were detected. Duplicate responses were identified by comparing contact information. Age was confirmed in the screener and main survey. Initially, however, this age check did not perform as intended. Subsequent data cleaning revealed that 4 individuals aged 13 years and 97 individuals aged 17 years had participated. These 101 youths were retained given that nothing suggested that their survey responses were otherwise invalid. For this investigation, 211 youths who completed the random digit dial survey were excluded because they did not provide answers to the lesbian, gay, bisexual, transgender–plus internalized stigma and pride measures, resulting in a final analytical sample of 4193 youths.

### Measures

The survey instrument and further details about measures are available upon request. Given the relative dearth of research available, our measures were guided by the minority stress model.^19,20,21,22^ This model posits that societal disadvantage leads to more stressful conditions and fewer resources, thereby resulting in negative health outcomes. Stressors among minority populations include bullying and peer aggression^21^ and reactions to these stressors, such as internalized stigma.^23,24^ Youths also are affected by generalized stressors. Positive exposures, such as social support, may buffer these stressors.^25,26^

Race and ethnicity were self-reported by youths. Individuals were asked if they were Hispanic. Response options for race were American Indian or Alaska Native, Asian, Black or African American, Native Hawaiian or Pacific Islander, White, mixed racial background, other race, and decline to answer. Individuals selecting other could specify their race. Those who responded mixed racial background were asked to name a group with which they most closely identified and could answer the previously listed groups, other, or decline to answer. Race and ethnicity were assessed because of their associations with racism and social marginalization that can contribute to health disparities.

#### Main Outcome

SV perpetration reflects 4 different behaviors.^27^ Youths were asked whether they had ever “kissed, touched, or done anything sexual with another person when that person did not want you to?” (ie, sexual assault). Given that the Bureau of Justice Statistics defines rape to include “psychological coercion as well as physical force,”^28^ youths were asked how often they ever tried but were not able to make someone have sex with them when they knew the individual did not want to (ie, attempted rape), made someone have sex with them when they knew the individual did not want to (ie, rape), or got someone to give in to sex with them when they knew the individual did not want to (ie, coercive sex).

#### Stratification Variable

Gender identity was measured with the following question and description: “What is your gender?” Youth were told that gender refers to cultural values (roles, behaviors, activities, and attributes) that a society associates with boys and girls. Gender also refers to how one identifies oneself. For many people, there isn’t a difference between these terms, but for some people, their gender identity is different from the sex they are assigned at birth. Response options were male; female; female-to-male (FTM)/transgender/trans man; male-to-female (MTF)/transgender/trans woman; genderqueer/nonbinary/pangender; other (specify), unsure; I don’t understand this question; and decline to answer. Individuals who endorsed male or female but reported a different sex assigned at birth, declined to answer either question about sex assigned at birth or gender identity, or said that their gender was other were asked, “Are you of transgender experience?”

Sex assigned at birth was measured by asking youths to report the sex on their original birth certificate. Those who endorsed male or female gender and reported the same sex assigned at birth or reported a different sex assigned at birth and did not endorse being of transgender experience were categorized as cisgender boys and girls (3282 individuals). Those who endorsed FTM/transgender/trans man or MTF/transgender/trans woman were together categorized as transgender boys and girls (329 individuals). Those who endorsed genderqueer/nonbinary/pangender (448 individuals), other (62 individuals), or unsure (15 individuals) and those who endorsed being of transgender experience (57 individuals) were categorized as nonbinary youths (582 individuals). Six additional youths declined to answer, and 11 individuals did not understand the gender question. These youths were coded as the majority category (ie, cisgender) and included in the 3282 cisgender boys and girls listed previously.

### Exposures, Guided by the Minority Stress Model

#### Other Indicators of Marginalized Status

Female gender was reported as described above. Youths who reported any sexual identity other than heterosexual were coded as sexual minority youths.

#### Stressors Associated With Being Marginalized

The Transgender Identity Survey (8 items; score range, 8-40; α = 0.92) was used to measure internalized transphobia.^29^ The 8-item Internalized Stigma Scale (score range, 8-32)^30,31^ was used to measure internalized homophobia (eg, α = 0.91 for those assigned male sex at birth and α = 0.87 for those assigned female sex at birth).

#### Factors Associated With Protection

Social support was measured by the Multidimensional Scale of Perceived Social Support (9 items, with friend and special person subscales; score range, 9-63; α = 0.91).^32^ Identity affirmation was measured with the 3-item subscale from the Lesbian, Gay, and Bisexual Identity Scale (score range, 3-15; α = 0.94).^33,34^

#### Generalized Stressors

Lifetime experience of trauma unrelated to SV (9 items),^35,36^ experience of bullying in the prior year (6 items; α = 0.66),^37^ any history of experience of serious SV (using the same language as described previously for SV perpetration), and experience of sexual harassment in the prior year (9 items; α = 0.90)^38,39^ were measured (all with responses of ever [1] or never [0]). These measures reflected individuals’ exposure to generalized stressors.

#### Environmental Factors

Environmental factors included low income and exposure to community violence. Low income was defined as “lower than the average family.” Exposure to community violence was defined as ever seeing someone attacked on purpose.^40^

#### General Factors

General risk factors associated with SV were also measured. These included past-year exposure to violent and nonviolent pornography,^10^ lifetime history of exposure to spousal abuse,^40^ attitudes accepting of rape (3 items) by boys (α = 0.89) and girls (α = 0.93),^41^ sexual dominance (12 items; score range, 0-60; α = 0.90),^42^ past-year alcohol use (with responses of ever [1] or never [0]),^43^ past-year friends’ involvement in SV (4 items; responses of ever [1] or never [0]; α = 0.82), and past-year aggressive behavior (4 items; responses of ever [1] or never [0]; α = 0.54).^44,45^

### Survey Process

Survey process indicators were additionally measured. These included self-reported honesty in answering survey questions and being alone when taking the survey.

### Statistical Analysis

Analyses were conducted with Stata statistical software version 14 (StataCorp). Hypothesis tests were 2-sided. Given the novelty of the research, a permissive *P* value < .20 or odds ratio (OR) > 2.0 were used for consideration at the bivariate level and inclusion in each multivariate logistic regression model (see later section). Data were analyzed in November 2021 and March 2022.

Less than 5% of data were missing for variables except 1 item in the internalized homophobia scale (103 of 868 youths who were assigned male at birth [11.9%] and 266 of 1729 individuals who were assigned female at birth [15.4%]). Missing data were coded as no exposure or to the scale mean. There was 1 outlier: almost all (10 of 12) items on the sexual dominance scale had rates of missingness greater than 5%. For this scale, missing data were coded directly and included in the sum variable.

Rates of perpetration and experience of SV were first examined by gender using χ^2^ and *t* tests. Next, prevalence rates of measures reflective of the Minority Stress Model (independent variables) were examined, stratified by gender and SV perpetration status (dependent variable) and quantified with logistic regression. We used backward and forward stepwise model-building procedures to identify a parsimonious model of the characteristics that retained statistical significance within the context of other statistically significant characteristics. Only youth characteristics associated with SV perpetration at the bivariate level were tested for inclusion. We estimated 3 parsimonious models: 1 for cisgender boys and girls, 1 for transgender boys and girls, and 1 for nonbinary youths. Survey process measures and girl gender for cisgender and transgender youths were retained in all parsimonious models irrespective of statistical significance.

## Results

Among 4193 youths (mean [SD; range] age, 14.8 [0.7; 13-17] years), 3282 participants (78.3%) identified as cisgender girls or boys; 582 participants (13.9%) identified as nonbinary, questioning, or unsure about their gender or being of transgender experience; and 329 participants (7.9%) identified as transgender boys or girls. Only 34 participants identified as transgender girls, so we were unable to disaggregate gender by boys and girls; all subsequent analyses examined 3 groups of youths: cisgender boys and girls, transgender boys and girls, and nonbinary youths. One in 5 participants was Hispanic (99 individuals [21.9%]), and there were 88 American Indian or Alaska Native individuals (2.1%), 170 Asian individuals (4.1%), 232 Black or African American individuals (5.5%), 17 Native Hawaiian or Pacific Islander individuals (0.4%), 2801 White individuals (66.9%), 125 individuals who declined to answer (3.0%), 574 individuals with mixed racial background (13.7%), and 180 individuals with other race (4.3%). Table 1 shows additional demographic characteristics.

**Table 1.** Youth Characteristics by Gender Identity and Sexual Violence Perpetration

Characteristic	Total (N = 4193)	Cisgender boys and girls			Transgender boys and girls			Nonbinary youths		
		SV perpetration, No. (%)		*P* value^a^	SV perpetration, No. (%)		*P* value^a^	SV perpetration, No. (%)		*P* value^a^
		No (n = 3039)	Yes (n = 243)		No (n = 307)	Yes (n = 22)		No (n = 548)	Yes (n = 34)	
Stressor associated with being marginalized										
Transphobia (n = 824; score range, 8-40), mean (SD)	25.6 (9.0)	NA^b^	NA^b^	NA	28.3 (8.5)	30.0 (7.9)	.37	23.6 (8.8)	24.4 (10.1)	.65
Homophobia (n = 2597; score range, 8-32), mean (SD)	16.6 (5.8)	17.0 (6.0)	17.4 (5.7)	.51	16.3 (5.2)	15.2 (5.3)	.36	15.3 (5.2)	15.6 (5.9)	.82
Generalized stressor										
Traumatic experiences (childhood adversity)	3726 (88.9)	2657 (87.4)	220 (90.5)	.16	286 (93.2)	21 (95.5)	.68	509 (92.9)	33 (97.1)	.35
Bullying	1194 (28.5)	739 (24.3)	83 (34.2)	.001	138 (45.0)	11 (50.0)	.65	210 (38.3)	13 (38.2)	.99
Peer aggression	1828 (43.6)	1254 (41.3)	131 (53.9)	<.001	156 (50.8)	11 (50.0)	.94	255 (46.5)	21 (61.8)	.08
Serious sexual violence	1824 (43.5)	1140 (37.5)	138 (56.8)	<.001	181 (59.0)	15 (68.2)	.39	327 (59.7)	23 (67.7)	.36
Sexual harassment (any)	3177 (75.8)	2246 (73.9)	195 (80.3)	.03	246 (80.1)	19 (86.4)	.48	439 (80.1)	32 (94.1)	.04
Other marginalized status										
Girl	1869 (44.6)	1724 (56.7)	109 (44.9)	<.001	33 (10.8)	1 (4.6)	.36	NA^b^	NA^b^	NA
Sexual minority identity	2547 (60.7)	1527 (50.3)	142 (58.4)	.01	297 (96.7)	21 (95.5)	.75	526 (96.0)	34 (100.0)	.23
Factor associated with protection										
Social support (range, 9-63), mean (SD)	48.1 (11.9)	48.2 (11.9)	45.8 (12.7)	.003	48.6 (11.9)	51.1 (12.0)	.34	48.3 (11.6)	46.8 (10.4)	.47
Pride in sexual or gender minority status (n = 2627; range, 3-15), mean (SD)	12.2 (3.0)	11.9 (3.0)	11.5 (3.5)	.16	12.4 (2.9)	12.3 (2.9)	.93	13.1 (2.6)	13.2 (2.3)	.74
Environment										
Low income	983 (23.4)	640 (21.1)	59 (24.3)	.24	99 (32.3)	5 (22.7)	.35	168 (30.7)	12 (35.3)	.57
Exposure to community violence	2626 (62.6)	1844 (60.7)	158 (65.0)	.18	210 (68.4)	17 (77.3)	.39	371 (67.7)	26 (76.5)	.29
General risk factor associated with SV										
Rape attitudes for boys who use violence (score range, 3-15), mean (SD)	3.4 (1.3)	3.4 (1.3)	3.9 (1.8)	<.001	3.3 (1.1)	3.2 (0.9)	.76	3.2 (0.9)	3.1 (0.4)	.33
Rape attitudes for girls who use violence (score range, 3-15), mean (SD)	3.5 (1.5)	3.6 (1.5)	4.1 (2.1)	<.001	3.2 (1.0)	3.2 (0.9)	.77	3.3 (1.1)	3.1 (0.4)	.24
Sexual dominance (score range, 0-60), mean (SD)	33.3 (12.1)	33.4 (11.9)	38.3 (11.0)	<.001	31.8 (12.2)	34.6 (11.1)	.29	31.6 (12.8)	34.7 (9.6)	.16
Consumption of pornography										
None	1231 (29.4)	941 (31.0)	36 (14.8)	<.001	78 (25.4)	5 (22.7)	.44	167 (30.5)	4 (11.8)	.05
Nonviolent	2186 (52.1)	1604 (52.8)	140 (57.6)		156 (50.8)	14 (63.6)		254 (46.4)	18 (52.9)	
Violent	776 (18.5)	494 (16.3)	67 (27.6)		73 (23.8)	3 (13.6)		127 (23.2)	12 (35.3)	
Exposure to spousal abuse	880 (21.0)	587 (19.3)	63 (25.9)	.01	87 (28.3)	11 (50.0)	.03	125 (22.8)	7 (20.6)	.76
Exposure to friends involved in SV	1116 (26.6)	717 (23.6)	87 (35.8)	<.001	110 (35.8)	8 (36.4)	.96	181 (33.0)	13 (38.2)	.53
Past-year alcohol use	1396 (33.3)	967 (31.8)	100 (41.2)	.003	113 (36.8)	11 (50.0)	.22	189 (34.5)	16 (47.1)	.14
Any aggressive behavior	1937 (46.2)	1367 (45.0)	140 (57.6)	<.001	141 (45.9)	14 (63.6)	.11	254 (46.4)	21 (61.8)	.08

Abbreviations: NA, not applicable; SV, sexual violence.

^a^ *P* values reflect *t* tests for continuous measures and χ^2^ tests for categorical measures.

^b^ No youths in this category.

### Prevalence Rates of Perpetration and Experience of SV by Gender Identity

The odds of SV perpetration were not significantly different for transgender boys and girls (OR, 0.90; 95% CI, 0.57-1.41; *P* = .64) or nonbinary youths (OR, 0.78; 95% CI, 0.54-1.12; *P* = .18) compared with cisgender boys and girls (Table 2). In contrast, transgender boys and girls (OR, 2.31; 95% CI, 1.83-2.91; *P* < .001) and nonbinary youths (OR, 2.37; 95% CI, 1.98-2.83; *P* < .001) were more than 2-fold as likely as cisgender boys and girls to report experiencing SV.

**Table 2.** Bivariate Comparisons of SV Involvement by Gender Identity

Type of SV	Cisgender boys and girls (n = 3282)	Transgender boys and girls (n = 329)	OR (95% CI)^a^	Nonbinary youths, No. (%) (n = 582)	OR (95% CI)^a^
SV perpetration					
Any	243 (7.4)	22 (6.7)	0.90 (0.57-1.41)	34 (5.8)	0.78 (0.54-1.12)
Sexual assault	171 (5.2)	18 (5.5)	1.05 (0.64-1.74)	30 (5.2)	0.99 (0.66-1.47)
Attempted rape	66 (2.0)	4 (1.2)	0.60 (0.22-1.66)	6 (1.0)	0.51 (0.22-1.18)
Rape	16 (0.5)	3 (0.9)	1.88 (0.54-6.48)	1 (0.2)	0.35 (0.05-2.65)
Coercive sex	41 (1.3)	2 (0.6)	0.48 (0.12-2.01)	5 (0.9)	0.68 (0.27-1.74)
SV experience					
Any	1278 (38.9)	196 (59.6)	2.31 (1.83-2.91)	350 (60.1)	2.37 (1.98-2.83)
Sexual assault	1105 (33.7)	181 (55.0)	2.41 (1.92-3.03)	326 (56.0)	2.51 (2.10-3.00)
Attempted rape	557 (17.0)	96 (29.2)	2.02 (1.56-2.60)	156 (26.8)	1.79 (1.46-2.20)
Rape	247 (7.5)	68 (20.7)	3.20 (2.38-4.31)	100 (17.2)	2.55 (1.98-3.28)
Coercive sex	350 (10.7)	59 (17.9)	1.83 (1.35-2.48)	112 (19.2)	2.00 (1.58-2.52)

Abbreviations: OR, odd ratio; SV, sexual violence.

^a^ Cisgender boys and girls were the reference group for logistic regression models; 10 models were estimated, 1 for each type of SV perpetration and experience.

### Factors Associated With SV Perpetration by Gender Identity

Characteristics retained in parsimonious logistic regression models of youth characteristics are shown in Table 3. Results are stratified by and estimated within each gender.

**Table 3.** Characteristics Associated With Odds of SV Perpetration by Gender Identity^a^

Characteristic	Cisgender boys and girls (n = 3282)		Transgender boys and girls (n = 329)		Nonbinary youths (n = 582)	
	aOR (95% CI)	*P* value	aOR (95% CI)	*P* value	aOR (95% CI)	*P* value
Generalized stressor						
Serious sexual violence	1.83 (1.36-2.45)	<.001	NA	NA	NA	NA
Sexual harassment	NA	NA	NA	NA	2.93 (0.68-12.69)	.15
Peer violence	1.26 (0.95-1.67)	.11	NA	NA	NA	NA
Bullying	1.26 (0.94-1.70)	.12	NA	NA	NA	NA
Other marginalized status						
Girl gender	0.67 (0.50-0.90)	.008	0.44 (0.06-3.41)	.43	NA	NA
Sexual minority identity	1.20 (0.91-1.59)	.20	NA	NA	NA	NA
Factor associated with protection			NA	NA	NA	NA
Social support^b^	0.99 (0.98-1.00)	.007	NA	NA	NA	NA
General risk factor associated with SV						
Rape attitude for boys perpetrating SV^b^	1.15 (1.07-1.25)	<.001	NA	NA	NA	NA
Sexual dominance^b^	1.03 (1.01-1.04)	<.001	NA	NA	NA	NA
Consumption of pornography						
None	1 [Reference]	NA	NA	NA	1 [Reference]	NA
Nonviolent	1.56 (1.05-2.31)	.03	NA	NA	2.39 (0.78-7.30)	.13
Violent	1.86 (1.19-2.92)	.007	NA	NA	3.07 (0.95-9.93)	.06
Exposure to spousal abuse	NA	NA	2.29 (0.95-5.54)	.07	NA	NA
Exposure to friends involved in SV	1.43 (1.06-1.93)	.02	NA	NA	NA	NA
Any aggressive behavior	NA	NA	1.87 (0.75-4.65)	.18	1.61 (0.78-3.32)	.20
Survey process measure						
Dishonesty in responding	1.10 (0.47-2.60)	.82	NA^c^	NA	2.00 (0.24-16.87)	.53
Not alone when completing survey	0.92 (0.66-1.28)	.61	1.17 (0.47-2.93)	.73	0.74 (0.33-1.64)	.46

Abbreviations: aOR, adjusted odds ratio; NA, not applicable; SV, sexual violence.

^a^ Parsimonious logistic regression models built stepwise forward and backward. Variables with OR > 2.0 or *P* < .20 were retained, except for survey process variables, and girl gender for cisgender and transgender youths, which were forced into the final model irrespective of statistical significance.

^b^ Per unit increase in score.

^c^ All 5 youths who reported dishonesty did not report perpetration. This measure was therefore excluded for estimations.

#### Transgender Boys and Girls

Transgender youths who had ever been exposed to spousal abuse (adjusted OR [aOR], 2.29; 95% CI, 0.95-5.54; *P* = .07) were more likely to report SV aggression, holding other statistically significant factors equal, compared with transgender boys and girls who did not perpetrate SV. This was also the case for transgender youths who engaged in aggressive behavior in the prior year (aOR, 1.87; 95% CI, 0.75-4.65; *P* = .18).

#### Nonbinary Youths

Among otherwise-similar non-binary youths, those who had ever experienced sexual harassment were almost 3-fold as likely as those who had not to report perpetrating SV (aOR, 2.93; 95% CI, 0.68-12.69; *P* = .15). Nonbinary youths who reported past-year exposure to violent pornography (aOR, 3.07; 95% CI, 0.95-9.93; *P* = .06) or nonviolent pornography (aOR, 2.39; 95% CI, 0.78-7.30; *P* = .13) were more likely than those not exposed to pornography to report using SV. Similar to transgender youths, among nonbinary youths, aggression in the prior year was also concurrently associated with SV aggression (aOR, 1.61; 95% CI, 0.78-3.32; *P* = .20).

#### Cisgender Boys and Girls

Among cisgender boys and girls, experience of SV (aOR, 1.83; 95% CI, 1.36-2.45; *P* < .001), experience of peer aggression (aOR, 1.26; 95% CI, 0.95-1.67; *P* = .11), and sexual dominance (aOR per unit increase in score, 1.03; 95% CI, 1.01-1.04; *P* < .001) were associated with higher odds of using SV. Positive attitudes for boys to engage in rape behavior (aOR per unit increase in score, 1.15; 95% CI, 1.07-1.25; *P* < .001) and exposure to pornography, including nonviolent (aOR, 1.56; 95% CI, 1.05-2.31; *P* = .03) and violent (aOR, 1.86; 95% CI, 1.19-2.92; *P* = .007) pornography, and to friends involved in SV (aOR, 1.43; 95% CI, 1.06-1.93; *P* = .02) were also associated with higher odds of SV perpetration. Being a girl was associated with lower odds (aOR, 0.67; 95% CI, 0.50-0.90; *P* = .008), and sexual minority identity was associated with higher odds (aOR, 1.20; 95% CI, 0.91-1.59; *P* = .20) of using SV. Additionally, as social support increased, the odds of SV perpetration decreased among otherwise similar cisgender boys and girls (aOR per unit increase in score, 0.99; 95% CI, 0.98-1.00; *P* = .007).

## Discussion

This cross-sectional study found that gender minority youths were more likely to experience SV but equally likely to perpetrate SV compared with cisgender youths. Antitransgender sentiment and gender-based stigma may be associated with increased risk of SV among gender minority youths,^46,47^ but despite higher rates of experiencing SV, rates of sexual aggression were similar across genders. Our findings, therefore, run counter to historical stereotypes of sexual deviancy of gender minorities.^48^

High rates of trauma experiences for youths who perpetrated SV were notable, particularly so for transgender boys and girls, as well as nonbinary youths. For both groups, all but 1 youth reported adversity. This serves as an important reminder that youths who use aggression are often trying to navigate trauma of their own and may likely benefit from empathetic rather than punitive intervention.

Cisgender girls were significantly less likely than cisgender boys to report SV perpetration; we found similar patterns for transgender girls vs transgender boys. Moreover, findings for cisgender youths suggested that norms that perpetuate toxic masculinity were associated with SV perpetration: rape attitudes condoning boys’ perpetration and sexual dominance. These differences were not noted for transgender boys or girls or nonbinary youths. These findings suggest that prevention programs may need to address culturally asserted gender roles, particularly with cisgender boys and girls, but perhaps less so with transgender boys and girls and nonbinary youths, who may be actively contending with these gender norms or have already considered and rejected these stereotypes. Being gender inclusive is not equivalent to being gender neutral. Prevention programs that are challenging gender and sexuality norms should be evaluated for their relevance to gender minority youths.

Our findings suggest that gender minority youths faced stressors unique to their experience as transgender boys and girls and nonbinary youths and that these stressors may help to explain their odds of perpetration compared with other gender minority youths who did not use SV. For example, nonbinary youths who had experienced sexual harassment were more likely than those who had not to report using SV. Nonbinary youths may face discrimination-related harassment, which may help explain why sexual harassment is a factor associated with sexual aggression among these youths but not among cisgender youths. Previous studies suggest that sexual and gender minority individuals who internalize stigma-related beliefs may be more likely to perpetrate violence in general^49^ and SV specifically against their sexual and gender minority partners.^50,51^

### Future Directions

Sex and gender are inexorably linked, but assuming that body parts or sex assigned at birth are associated with greater changes in outcomes than gender identity when examining SV may be misguided,^52^ particularly given how social and structural expectations shape gender roles.^53^ Indeed, as suggested by characteristics identified in multivariate models, SV may reflect cultural factors, such as the need to dominate, for some but not all genders. Nonetheless, sexual organs are, by definition, involved in SV, a particularly important point during adolescence, when youths become more aware of their sexual selves. For gender minority youths, this can also be a time of trauma, as individuals begin to develop secondary sexual characteristics (eg, breast development) that may render it impossible to ignore dissonance between one’s body and one’s gender. Persistent gender dysphoria is also associated with increased risk of enacted stigma among gender minority youths.^54,55^ While older gender minority adolescents and young adults may be undergoing gender-affirming care, including hormone therapy and surgery, adolescents aged 14 to16 years may lack access to these care options. Moreover, gender expression is often policed, particularly for transgender girls. Future research should strive to better reflect this complexity analytically.

### Limitations

This study has several limitations. The cross-sectional design disallows causal inference. Relatedly, developmental timing may be associated with SV perpetration risk; different factors may be associated with greater changes in outcomes at different developmental time periods. Additionally, this study used past-year retrospective self-report measures; there are known confounds among reports of perpetration, adverse experiences, and same-source reporting bias.^56^ If we had been able to follow individuals prospectively using, for example, ecological momentary data, we may have obtained more specific data. It is impossible to know how this affected our results. Consistent with a 2020 study,^57^ we recruited significantly fewer transgender girls than any other group. Given this limitation, we were unable to disaggregate boys and girls for analyses. While perhaps a reflection of the true distribution in the population, findings may have been different if boys and girls were examined separately. Similarly, rates of generalized stressors in the current sample may be higher than those in other community samples. Associations between exposure and outcome should nonetheless generalize. Additionally, gender identity is diverse and fluid. The current measure was single response, disallowing youths to identify as both transgender and nonbinary, for example. Future studies should measure gender with multiple responses and endeavor to reflect this complexity in analyses.

## Conclusions

One might assume that higher rates of youths experiencing SV would be mirrored by higher rates of perpetration because relationships are dyadic. However, in this study, data suggest that rates of experiencing SV were higher but perpetration rates were similar for gender minority compared with cisgender youths. Perhaps this reflects differential reporting or more effective coping with SV trauma among gender minority youths, or perhaps antitransgender bias may lead some cisgender individuals to use SV as a way to punish and aggress against transgender boys and girls. Future research that examines gender identities of youths who have experienced and used SV may help to further inform these disparate findings.
